# Phage display for the detection, analysis, disinfection, and prevention of *Staphylococcus aureus*

**DOI:** 10.1002/SMMD.20220015

**Published:** 2022-12-23

**Authors:** Lei Tian, Kyle Jackson, Michael Chan, Ahmed Saif, Leon He, Tohid F. Didar, Zeinab Hosseinidoust

**Affiliations:** ^1^ Department of Chemical Engineering McMaster University Hamilton Ontario Canada; ^2^ School of Biomedical Engineering McMaster University Hamilton Ontario Canada; ^3^ Michael DeGroote Institute for Infectious Disease Research McMaster University Hamilton Ontario Canada; ^4^ Department of Mechanical Engineering McMaster University Hamilton Ontario Canada

**Keywords:** antimicrobial, bacteriophage, drug resistance, phage display, *Staphylococcus aureus*

## Abstract

The World Health Organization has designated *Staphylococcus aureus* as a global health concern. This designation stems from the emergence of multiple drug‐resistant strains that already account for hundreds of thousands of deaths globally. The development of novel treatment strategies to eradicate *S. aureus* or mitigate its pathogenic potential is desperately needed. In the effort to develop emerging strategies to combat *S. aureus*, phage display is uniquely positioned to assist in this endeavor. Leveraging bacteriophages, phage display enables researchers to better understand interactions between proteins and their antagonists. In doing so, researchers have the capacity to design novel inhibitors, biosensors, disinfectants, and immune modulators that can target specific *S. aureus* strains. In this review, we highlight how phage display can be leveraged to design novel solutions to combat *S. aureus*. We further discuss existing uses of phage display as a detection, intervention, and prevention platform against *S. aureus* and provide outlooks on how this technology can be optimized for future applications.

## Graphical abstract

Phage display is broadly used for detection, mapping, disinfection, and prevention of *Staphylococcus aureus.*

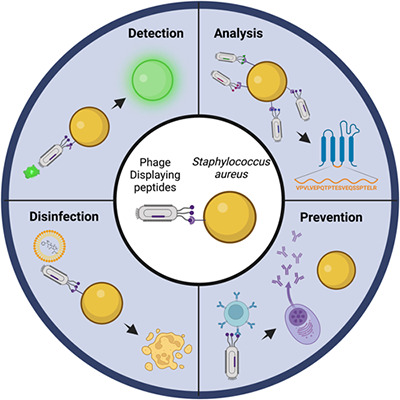

1

**Key points**

- This paper highlights how phage display can be leveraged to design novel solutions to combat *Staphylococcus aureus.*
- This paper discusses existing uses of phage display as a detection, intervention, and prevention platform against *S. aureus.*
- This paper provides outlooks on how this technology can be optimized for future applications.

## 2 INTRODUCTION

### 2.1 Staphylococcus aureus

*Staphylococcus aureus* is a gram‐positive, round‐shaped bacterium and has been associated in the development of many clinical manifestations that can either be community‐^1^, ^2^ or hospital‐acquired.^3^ To that end, it is considered a major human pathogen. *S. aureus* is commonly associated with many diseases like bacteremia, infective endocarditis, skin and soft tissue infections, osteomyelitis, septic arthritis, prosthetic device infections, pulmonary infections, gastroenteritis, meningitis, toxic shock syndrome, and urinary tract infections.^4^ Its pathogenic potential is highly dependent on the specific strain causing the infection,^5^, ^6^, ^7^ but in general, pathogenesis is induced either through invasive infections and/or toxin‐mediated diseases.^8^ Examples of toxins produced by *S. aureus* include hemolysin, leukotoxin, exfoliative toxin, enterotoxin, and toxic‐shock syndrome toxin‐1 (TSST‐1).^9^ What makes *S. aureus* difficult to treat is its capacity to evade the immune system. The secretion of enzymes like coagulase, proteases, and staphylokinase helps facilitate this evasion through the degradation of host signaling and metabolic pathways.^10^, ^11^ These enzymes simultaneously mediate *S. aureus*' invasion into host tissue, further exacerbating the infection.^12^, ^13^ Other modes of immune evasion include production of an antiphagocytic capsule,^14^, ^15^ sequestering of host antibodies or antigen masking by Protein A, biofilm formation, and intracellular survival.^16^, ^17^

Many of these infections are iatrogenic, meaning they are acquired through medical examination or treatment. *S. aureus* is notorious for contaminating medical devices.^18^, ^19^, ^20^ Given the increased usage of medical devices in our healthcare systems, this public health crisis is only expected to worsen. Concurrently, *S. aureus* bacteria are known to develop resistance to antibiotics that have historically been used to treat them.^21^, ^22^ The most consequential antimicrobial resistant (AMR) *S. aureus* is methicillin‐resistant *Staphylococcus aureus* (MRSA).^23^ In 2017, the World Health Organization published that MRSA is one of a growing list of AMR bacteria that require the urgent development of novel interventions.^24^ A recent meta‐analysis revealed that MRSA alone accounted for more than 100,000 deaths in 2019.^25^ With the declining rate of development of new antibiotic compounds,^26^ it is clear that novel solutions are urgently needed.

### 2.2 Phage display

Bacteriophages, also known as phages, are natural bacterial viruses, which are widely used as antimicrobials and building blocks.^27^, ^28^, ^29^, ^30^ Phage display is a popular laboratory technique that is used to study protein–protein, protein–peptide, and protein–DNA interactions.^31^, ^32^, ^33^ Protein sequences are encoded into the phage coat protein, which results in the phage “displaying” the protein on the surface of the virion.^32^ The resulting expressed protein can then be evaluated for binding capacity to other proteins, peptides, or DNA sequences to understand the interactions between the proteins and the molecules. This process is known as in vitro selection and can be used to screen a large library of proteins against specific targets.^34^ *Escherichia coli* filamentous bacteriophages such as f1, fd, and M13 are commonly used for phage display with most peptides being displayed at phage proteins pIII and pVIII.^35^ T4, T7, and lambda phages have also been used for phage display purposes.^35^

**FIGURE 1:**
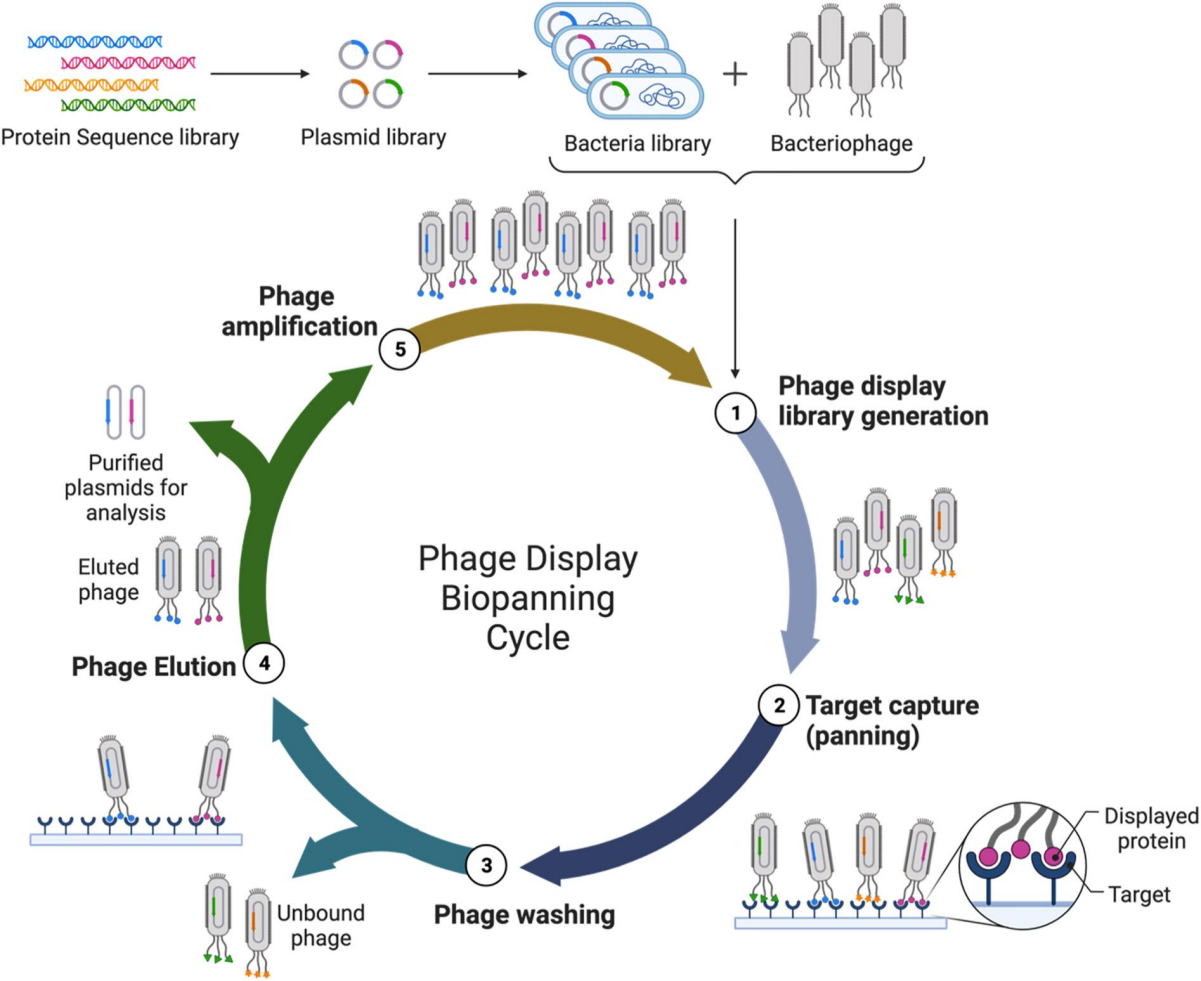
Phage Display Biopanning Cycle. Step 1, phage display libraries are prepared. Step 2, initial capture assessment of target performed. Step 3, the unbound phage washed off the target substrate. Step 4, the bound phage is eluted from the target substrate. Step 5, viral amplification of the eluted phage. Steps 2 through 5 repeated to determine high‐affinity phage display peptides.

Phage display was introduced in 1985 by George P. Smith, an American biologist.^36^ His work in developing phage display techniques ultimately awarded him the Nobel Prize in Chemistry in 2018. His 1985 work was the first report case demonstrating the display of external peptides on the surface of filamentous bacteriophages. He did this by fusing the virus’s capsid protein to a single peptide within a collection of sequences.^36^ Biopanning, an affinity selection technique critical to phage display, was later introduced in 1988 by Stephen Parmley and George Smith.^37^ In short, biopanning is the technique used to identify peptide sequences that interact strongly with a given target. It is also a technique used to select for high‐affinity antibodies. Biopanning has five major steps in its peptide selection process (Figure 1). Briefly, phage display libraries must first be prepared. This is followed by a capture step that involves the conjugation of the phage display library to the desired target; this step is known as panning. Next, a washing step is performed to remove the unbound phage display virions from the target molecule. Finally, an elution step is performed to remove the bound phages from the target molecule. These phages are then amplified using the appropriate bacterial vector. Steps 2 through 4 and viral amplification are then repeated several times to obtain highly specific peptide sequences for the given molecular target.^31^

### 2.3 Applications of phage display for *S. aureus* infections

The applications for phage display technology are numerous.^31^, ^38^, ^39^ Phage display is commonly used in protein engineering. For this reason, the technique can be incredibly useful for drug discovery and development. A common application of this technique has been employed to develop new treatments for cancer either through identifying tumor antigens or by expressing specific epitopes that could elicit strong immunological responses.^38^, ^40^

**FIGURE 2:**
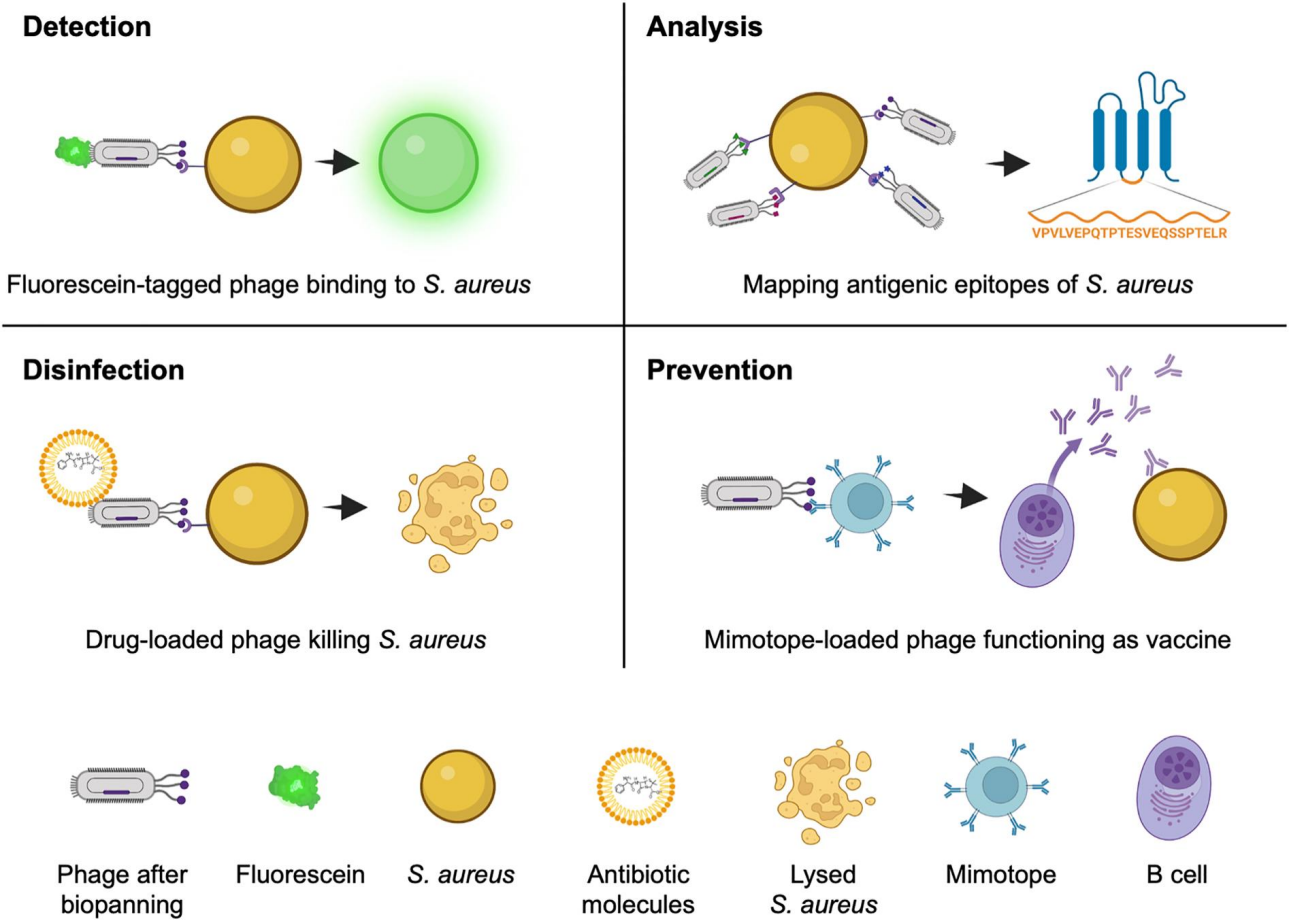
Main four application directions of phage display on *Staphylococcus aureus*, including detection, analysis, disinfection, and prevention.

Given the increasing burden of *S. aureus* on our healthcare systems, phage display is uniquely positioned to support efforts in counteracting its consequential impact. Similar to the phage display’s historical use of targeting tumor cells, there are several applications in which the technique can directly address the prevalence of *S. aureus* (Figure 2). First, phage display may serve as a cost‐effective biosensing alternative for the detection of microbes on surfaces or in bodily fluids. Phage display libraries can be designed in such a way that they may target whole cells,^41^, ^42^, ^43^, ^44^ cell surface proteins,^45^, ^46^, ^47^ or secreted virulence factors.^48^, ^49^, ^50^, ^51^ An advantage of phage display proteins is that when acting as biosensing tools, they do not necessarily require a substrate.^46^, ^50^ Conversely, if a substrate is preferred for a specific application, the platform is flexible enough so that it can be integrated onto two‐dimensional^45^ as well as three‐dimensional substrates.^41^, ^42^, ^43^, ^44^, ^47^

Another application of phage display is to help develop strategies to ameliorate the pathogenic tendencies of *S. aureus* through the mapping of different cell sites.^52^, ^53^, ^54^, ^55^, ^56^, ^57^, ^58^, ^59^ This application may serve several functions, including the identification of novel drug targets,^52^, ^53^ development of novel drug candidates,^54^, ^55^ or the display of specific antigens to work as a vaccine that elicits a strong immunological response^48^, ^60^, ^61^, ^62^, ^63^, ^64^, ^65^, ^66^, ^67^ (Figure 2). Furthermore, because phage display proteins have the capacity to bind strongly to their target receptors with high specificity, the platform can be used for the development of site‐specific drug carriers, potentially enhancing the therapeutic effect of a given compound.

**FIGURE 3:**
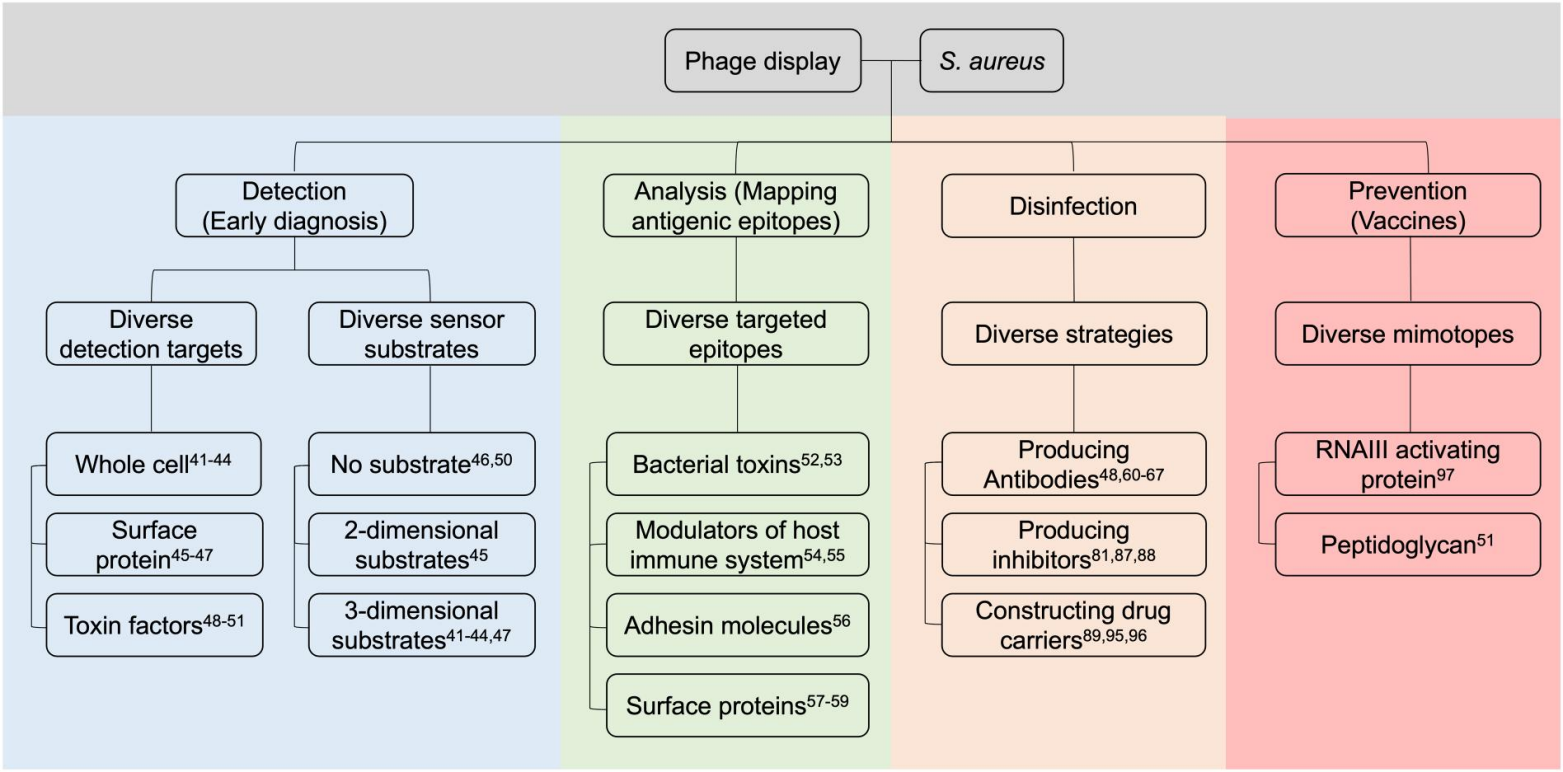
Summary of diverse applications of phage display on the detection,^41^, ^42^, ^43^, ^44^, ^45^, ^46^, ^47^, ^48^, ^49^, ^50^, ^51^ analysis,^52^, ^53^, ^54^, ^55^, ^56^, ^57^, ^58^, ^59^ disinfection,^48^, ^60^, ^61^, ^62^, ^63^, ^64^, ^65^, ^66^, ^67^, ^81^, ^87^, ^88^, ^89^, ^95^, ^96^ and prevention^51^, ^97^ of *Staphylococcus aureus*.

In this review, we focus on discussing how phage display can be used as a powerful tool to combat the emerging threat of *S. aureus* as a multidrug‐resistant bacterium. In addition to presenting the literature on how phage display can be used to identify the presence of *S. aureus*, we highlight how phage display is being used to design novel prevention and intervention strategies that can be deployed to neutralize the bacterium (Figure 3).

## 3 DETECTION OF *S. AUREUS* USING PHAGE DISPLAY

### 3.1 Phage‐displayed peptides targeting different components of *S. aureus*

When using phage display, investigators attempt to identify selected peptides to bind to a specific molecular target. In the case of *S. aureus*, the main associated targets include the whole cell,^41^, ^42^, ^43^, ^44^ surface proteins,^45^, ^46^, ^47^ and toxin factors^48^, ^49^, ^50^, ^51^ of the bacteria. Researchers have used peptides as effective probes in detecting these targets. The study methods and findings are outlined below (Table 1).

**TABLE 1:** Identification of peptide sequences that have a binding affinity for the different components of *S. aureus* cells

Target	Name	Sequence of peptides screened from phage display
Whole cell	N/A	GLHTSATNLYLH^41^ RVRSAPSSS^42^, ^43^ LHVNPGLQTLQG^44^ GIGKFLHSAGKFGKAFVGEIMKS^44^
Surface proteins	Outer membrane surface 8 kDa protein Protein A	VPHNPGLISLQG^46^ RVRSAPSSS^45^ GQTTLTTS^47^
Toxin factors	Staphylococcal enterotoxin B (SEB)	WHKAPRAPAPLL^50^ MNLHDYHRLFWY^49^ MQVQLQESGEAQAGGSLRLSCTASGYTGWGWFRQAPGKEREGVAMVSGIGAAGTYTLSRLPRGPIHHLPRQRQEHGVPKTTPICKTTNLRTRPSITVRQILLPCVGLLPGNINGYDYWGQGTQVTVSS^48^ MQVQLQESGEAQAGGESLRLSCTASGYTGWGWFRQAPGKEREGVAMVSGIGAAGTYTLSRLPRGPIHHLPRQRQEHGVPKTTPICKTTNLRTRPSITVRQILLPCVGLLPGNINGYDYWGQGTQVTVSS^48^

#### 3.1.1 *S. aureus* whole cell

Phage libraries are often screened against a definite target and selected based on the binding affinity of the associated peptides. Though there are peptides that demonstrate an affinity for *S. aureus* cells, it occasionally remains unclear as to the exact target that is being selectively bound on the bacterial cell. Many studies have used phage display to detect *S. aureus* as a whole and used that information to determine relative concentration ranges and lowest detection limits of the bacterial cells.^41^, ^42^, ^43^, ^44^ In the end, however, investigators were not able to conclude what the peptides were selectively binding to on *S. aureus* cells. Nonetheless, whole cell detection methods offer a cost‐effective and efficient diagnostic tool that can be of use in different detection platforms that do not require significant optimization.

#### 3.1.2 Bacterial surface proteins

Microbial interactions between bacteria and its host are mediated by bacterial cell surface proteins and host receptor molecules.^68^ *S. aureus* may express these surface proteins as virulence factors^69^; however, specific mechanisms remain poorly understood. There are still many surface markers of *S. aureus* that remain undiscovered. Investigating and discovering these novel markers may allow for the development of effective detection of the bacterium. Consequently, phage display technology can be used as an efficient method to select from a variety of peptides that demonstrate high binding affinity and specificity to the surface markers of *S. aureus* cells. This was first introduced by Rao et al. where a synthetic peptide with a high specific binding potential to *S. aureus* was discovered.^46^ The investigators wanted to further understand how the peptide they had designed had specifically bound to the *S. aureus*. To achieve this, they probed lysates of the *S. aureus* strain with the displayed peptide. In doing so, the peptide was most prominently bound to a 60 kDa protein of *S. aureus* in addition to 40 and 50 kDa proteins. Similarly, another study conducted by De Plano et al.^45^ found that a phage clone displaying a peptide exhibited specific binding to the *S. aureus* cell surface with a 78 kDa protein as the recognized target. As such, the protein is believed to be critical for cell division of *S. aureus*.

There are many proteins expressed on the surface of *S. aureus* cells that are considered virulence factors and may activate or alter the host immune response. Proteins such as protein A were speculated by Liu et al.^47^ to be the binding target of their study. The peptide system isolated from phage display covered a majority of the cell surface of *S. aureus* cells, and given that protein A is an important component of the cell wall of an *S. aureus* cell, it is believed to be complementary to the developed peptide.

#### 3.1.3 Toxic factors

As a dangerous and versatile pathogen, *S. aureus* possesses a variety of virulence factors that allow it to cause a multitude of severe diseases. One subset of virulence factors is staphylococcal enterotoxins, which are potent bacterial super antigens with toxic effects that directly interfere with host cells.^8^ Enterotoxins are commonly known for their pyrogenic effects and interference with intestinal function; hence, they are mainly associated with staphylococcal food poisoning in humans.^70^ While there are many types of staphylococcal enterotoxins, staphylococcal enterotoxin B (SEB) induces one of the most profound toxic effects because of its ability to mount a massive host immune response, leading to a cytokine storm and subsequent acute toxic shock.^71^ Effective and reliable detection methods must be carried out in identifying SEB during clinical diagnosis and food analysis. Immunological techniques, such as enzyme‐linked absorbent assay (ELISA), are the most commonly used approaches; however, they are both time‐consuming and inconvenient.^72^ In addition, these assays use monoclonal antibody for detection in which they are of high cost and have continually become a major burden in the healthcare system.^48^

Alternative means to developing antibodies have recently been discovered with the use of antibodies from camelids. These types of antibodies are capable of antigen binding despite not having any light chains.^73^ Consequently, they are mostly known as a nanobody or camelid heavy chain antibody (VHH). VHH may render the use of traditional monoclonal antibodies obsolete as VHH has become more profound in numerous medical applications. To investigate its purpose with SEB, an SEB‐specific and sensitive camelid nanobody was produced using phage display.^48^ Two phage clones were found to have the highest affinity to SEB with one of them demonstrating a high specificity of 10^−9^ M. Phage display technology on its own provides an ideal pathway for detecting SEB with the screening of peptides that may prove to be highly SEB‐specific. In a study, the authors selected for SEB‐binding peptides and were able to discover three peptides from the library that demonstrated high binding affinities to SEB.^49^ The three peptides did not share a consensus sequence, and this demonstrates the variety of epitopes of the SEB molecule. The peptide with the highest binding affinity for SEB had a binding constant of 4.2 ± 0.7 × 10^5^/M, indicative of a strong binding capacity compared to an antibody with 1.6 ± 0.8 × 10^7^/M. Similarly, phage clones displaying peptides can be used to investigate its binding capacity to SEB. This allows for the whole phage to be fluorescently labeled and enhances the detection of bacterial cells. Goldman et al. selected phage clones that had an affinity for SEB, while possessing similar sequences in their amino terminus.^50^ Subsequently, the selected phage clones were fluorescently labeled with the dye Cy5 and they detected the lowest concentration of SEB.

### 3.2 Different detection substrates

Different methods of utilizing phage display peptides to detect *S. aureus* have been developed. Some researchers simply mix labeled peptides with *S. aureus* (nonsubstrate method),^46^, ^50^ while others use two‐dimensional^45^ or three‐dimensional substrates^41^, ^42^, ^43^, ^44^, ^47^ to integrate peptides. The detailed methods and corresponding detection sensitivity are illustrated below.

#### 3.2.1 Nonsubstrate detection methods

**FIGURE 4:**
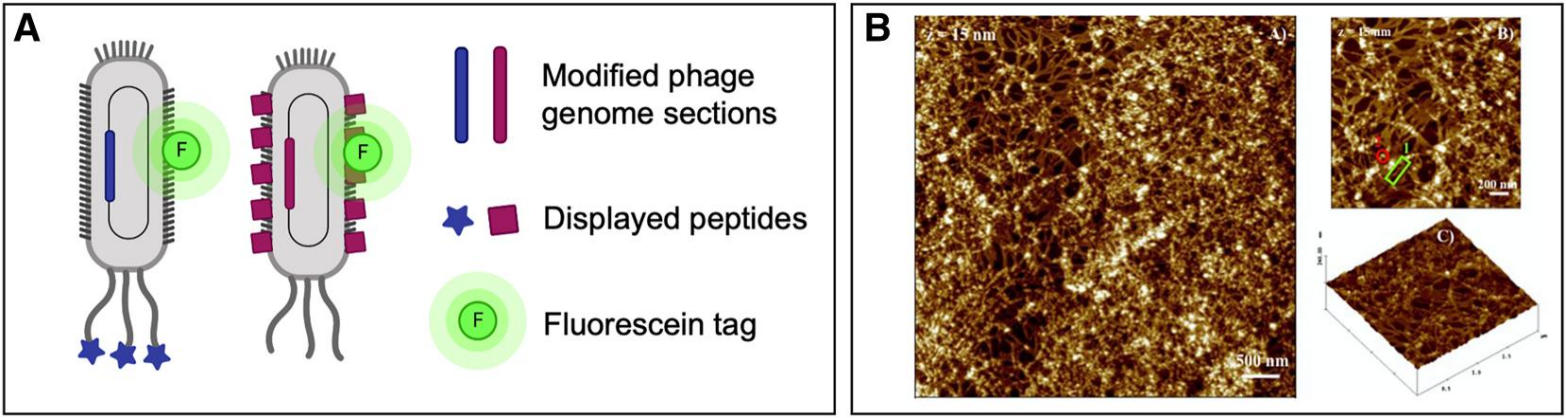
Detection of *Staphylococcus aureus* using phage‐displayed peptides with no substrate or two‐dimensional substrate. (A) No substrate: schematic image of free fluorescein‐tagged phages displaying certain peptides. (B) 2D substrate: AFM images of phage‐displayed peptides deposited onto the surface of mica substrates. Adapted with permission.^45^ Copyright 2017, Elsevier.

Conventional methods of phage display involve immobilizing the desired target and subsequently screening a phage library expressing different peptides. When peptides that bind to the molecular target with high affinity are found, they are isolated, processed, and further analyzed in future experiments.^49^ For instance, Rao et al. utilized a simplistic approach with regard to screening a phage display library where a peptide was found to have a binding affinity for *S. aureus* cells.^46^ The addition of a subtractive panning method helped to deplete phages against nontarget pathogenic bacteria, thereby efficiently reducing the number of peptides exhibiting nonspecific binding to *S. aureus*. The subtractive phage display approach proved to be effective in identifying peptides that bind selectively to *S. aureus* cells. To test diagnostic potential, the peptide of interest was conjugated with quantum dots and incubated with human platelets and a panel of bacteria.^46^ Through fluorometric analysis, the peptide was found to be highly specific for *S. aureus* with minimal cross‐reaction to other tested bacterial strains. The peptide had a detection limit of 100 colony forming unit per milliliter (CFU/ml).^46^ In a recent study, a Cy5 dye and a fiber optic biosensor were used to analyze the detected signal from the attachment of selected phage clones on a toxin secreted by *S. aureus* cells^50^ (Figure 4A). The complex was capable of detecting the lowest concentration of 1.4 ng/well. As such, these results demonstrate the possible development of phage‐based sensor reagents.

#### 3.2.2 Two‐dimensional substrates

Biosensors most often incorporate antibodies and proteins as ligands to probe specific molecular targets. With phage display as an emerging technique, it would be suitable to investigate the possibility of using it to develop a biosensor device. De Plano et al. investigated this approach by binding selected phage to a mica surface to form a biosensor that is selective for *S. aureus* cells^45^ (Figure 4B). After evaluating the mica‐physiosorbed phage, it was found that the complex was able to bind and capture approximately 50% of *S. aureus* cells within minutes and 90% of it after an hour of exposure. This study did not report a detection limit, but still demonstrates the promising potential in creating a lab‐on‐chip platform that is inexpensive, specific, and rapid in the detection of bacterial targets.

#### 3.2.3 Three‐dimensional substrates

**FIGURE 5:**
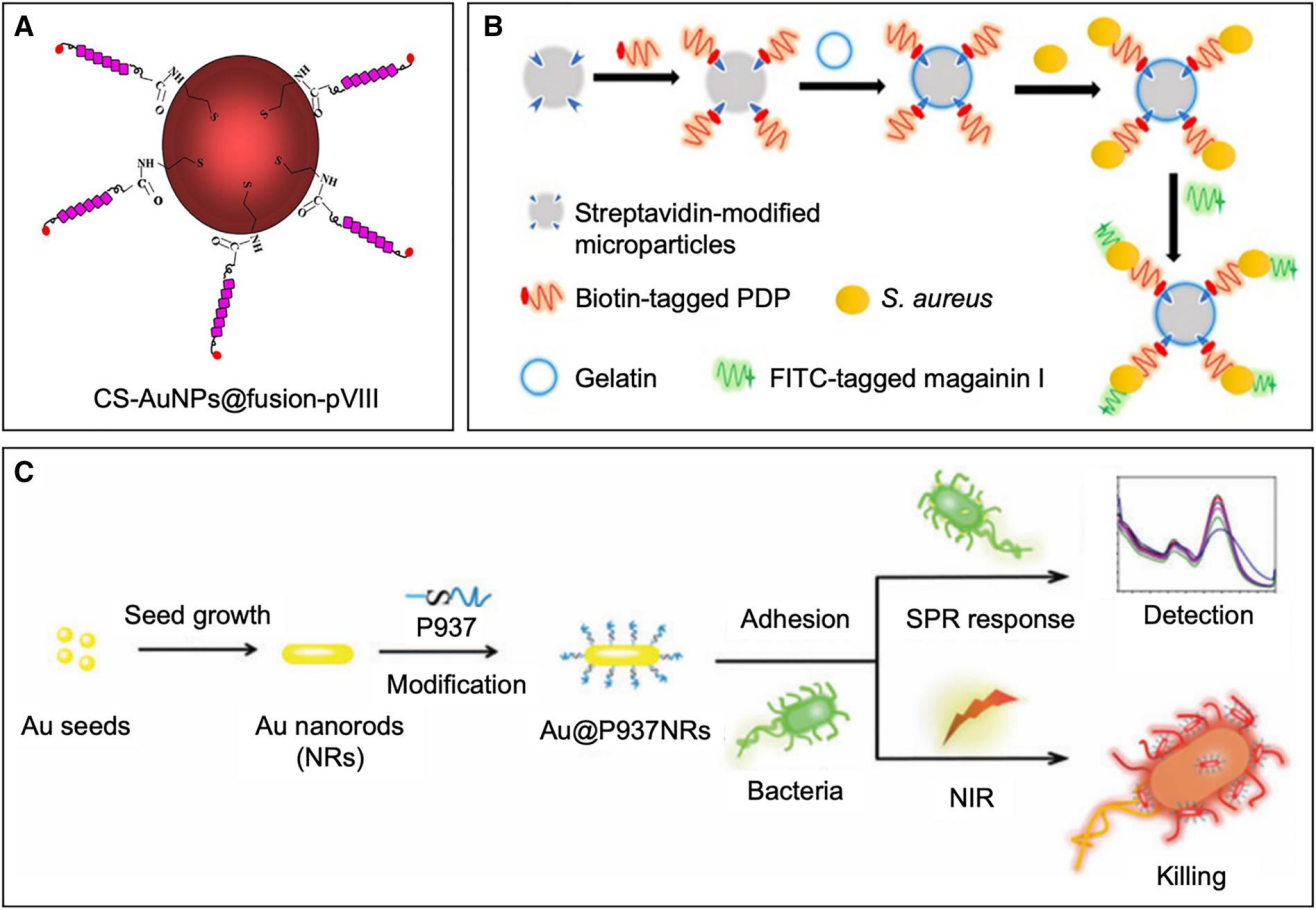
Detection of *Staphylococcus aureus* using phage‐displayed peptides with three‐dimensional substrates. (A) Schematic of a Au nanoparticle coated with phage‐displayed peptides. Adapted with permission.^47^ Copyright 2016, Elsevier. (B) Schematic image of sandwich fluorimetric detection of *S. aureus* using magnetic microparticles coated with phage‐displayed peptides. Adapted with permission.^44^ Copyright 2017, Springer Nature. (C) Schematic of Au nanorods coated with phage‐displayed peptides for detecting bacteria. Adapted with permission.^41^ Copyright 2018, Royal Society of Chemistry.

Nanoparticles exhibit many desirable physical and chemical properties, such as high stability, biocompatibility, nontoxicity, and surface modifiability, making them an ideal platform for detection systems.^74^ Through screening and selecting peptides via phage display, targeting ligands can be conjugated to nanoparticles, producing a complex biosensor capable of probing bacterial cells. This method was first introduced by Liu et al. where an *S. aureus*‐specific peptide demonstrating high specificity and affinity was used to functionalize cysteamine‐stabilized gold nanoparticles (CS‐AuNPs)^47^ (Figure 5). Consequently, the bifunctional nanoprobe (CS‐AuNPs@fusion‐pVIII) had increased recognition of the bacterial target along with improved selectivity of the gold nanoparticle sensors. After evaluation, this complex probe was shown to aggregate on the cell surface of *S. aureus* and was able to detect concentrations as low as 19 CFU/ml within 30 min, proving to be a sensitive, selective, and inexpensive method to rapidly detect bacteria.^47^

Likewise, gold nanorods share similar features with gold nanoparticles aside from their size difference. Using phage display, Chen et al. selected an adhesion peptide P937 with a high binding capacity to *S. aureus* and conjugated them with gold nanorods.^41^ The product was a dual‐functional peptide coupling gold nanoconjugate (Au@P937 NRs), which was shown to rapidly aggregate on the surface of *S. aureus* cells with a detection limit of 89 CFU/ml. Unlike nanoparticles, other gold nanostructures, such as nanorods, can be used as photothermal agents. Au@P937 NRs were not only capable of detection, but were also capable of killing bacterial pathogens through a gold‐based photothermal bacterial lysis method in 10 min under 808 nm laser irradiation.

Silicon nanoparticles (SiNPs) can also be used for detection methods and have shown recent success in identifying bacterial cells. De Plano et al. conjugated phage‐specific pVIII proteins displaying *S. aureus* targeting peptides onto silicon nanoparticles. These bioconjugates formed networks covering *S. aureus*, binding to cells within 30 min.^42^ No detection limit was reported. Even while functionalized, SiNPs retained their inherent fluorescence and could be used as fluorescent nanoprobes. Thus, biofunctionalized silicon‐based nanoparticles represent a novel avenue for developing rapid and specific diagnostic platforms for microorganisms.

Magnetic beads functionalized by phages hold potential as a detection tool that is cheap, highly sensitive, and specific in detecting bacteria. Without compromising the ability of phages to bind to *S. aureus* even after being conjugated onto magnetic beads, De Plano et al. demonstrated that this complex was capable of capturing a maximum of 60%–70% of *S. aureus* cells with a limit of detection of 10 CFU/7 mL.^43^ The phage magnetic complex allowed for the isolation of bacterial cells present in blood samples with subsequent micro‐Raman spectroscopy enabling the specific and efficient detection of bacteria. Unlike the aforementioned studies that involved a single peptide, Xiong et al. utilized a dual‐peptide‐recognition strategy for sandwich fluorometric detection.^44^ To maximize specificity in detecting *S. aureus* cells, two recognition peptides, one of which was selected from phage display, were functionalized onto magnetic particles. This sandwich biological complex exhibited an 86.4% capturing efficiency to *S. aureus* with a detection limit as low as 9 CFU/ml.

The present research demonstrates phage display technology as a promising approach to producing nanobodies, peptides, and phage clones that exhibit high specificity and sensitivity to all components of *S. aureus* cells. It should be noted that much of the platforms outlined above have been evaluated using homogenous solutions of bacteria, whereas in reality, biological environments are heterogenous. Despite this, these findings provide pathways for further enhancement in developing more complex detection methods, such as nanobody‐based detection systems and biosensor applications. Future work should focus on assessing and optimizing the capability of these platforms to detect *S. aureus* in heterogenous communities.

## 4 ANALYSIS: MAPPING ANTIGENIC EPITOPES OF *S. AUREUS* USING PHAGE DISPLAY

An epitope is described as the specific region of an antigen that binds to antibodies when an immune response is activated.^75^ Mimotopes are peptide sequences that mimic the structural features of an epitope, and similar to its respective counterpart, elicit an immune response.^76^ Mapping epitopes of *S. aureus* can not only help us understand the bacterial pathogenic mechanism,^77^ but also contribute to the mimotope design for novel vaccine development, which will be introduced later. Phage display technology plays an important role in the process of epitope mapping where peptides are screened to select for mimotopes.^56^ The process of mapping antigenic epitopes is different from screening peptides for *S. aureus* detection. In fact, epitope mapping applied an opposite working flow where antibodies of specific epitopes from *S. aureus* were firstly produced and isolated from animals.^56^

Phage display libraries are screened and panned against anti‐specific epitope antibodies to identify peptides as potential mimotopes. Recent work has identified *S. aureus* proteins responsible for the toxicity, infection, and modulation of the immune response of the bacterium (Table 2).^52^, ^53^, ^54^, ^55^ This helps to screen for peptides that mimic these antigenic epitopes and can be used as vaccine candidates in murine models and possibly to prevent infections. In addition, antigenic epitopes of adhesion molecules, surface proteins, and peptides have also been explored to understand molecular interactions between *S. aureus* and host cells (Table 2).^56^, ^57^, ^58^, ^59^ While this platform will enable the rapid identification of vaccine candidates, it does not alleviate the broader challenges associated with vaccine development.^78^ Doubtless, these combined findings suggest clinical translatability in using phage display to develop human vaccine candidates that protect against *S. aureus* infections.

**TABLE 2:** Identification of targeted epitopes on *S. aureus* cells using phage display

Types	Targeted epitopes
Bacterial toxins	Tryptophan regulated attenuation protein (TRAP)^52^ Staphylococcal enterotoxin B (SEB)^53^
Modulators of host immune system	Chemotaxis inhibitory protein (CHIPS)^54^ SSL6 and SEIX^55^
Adhesin molecules	ClfA^56^
Surface proteins	Glyceraldehyde‐3‐phosphate dehydrogenase C (GapC)^57^ Fibronectin‐binding protein A^58^ Extracellular fibrinogen‐binding protein (Efb)^59^

## 5 DISINFECTION OF *S. AUREUS* USING PHAGE DISPLAY

Historical progression of *S. aureus* treatment regimen has been guided by the need to overcome acquired resistances to formerly anti‐*Staphylococcal* drugs, including penicillin, sulfonamides, tetracyclines, among others.^79^ In the 1960s, methicillin‐resistant *S. aureus* isolates were discovered. More recently, strains resistant to vancomycin, deemed the “drug of last resort,” have emerged. Developing antimicrobials with new molecular targets or improving the efficacy of existing therapies will be crucial in maintaining our ability to treat complex clinical manifestations of *S. aureus* infection. By displaying a library of peptides of the surfaces of bacteriophages, phage display is a promising tool that can be used to screen, select, and synthesize specific and high‐affinity molecules that can broaden our current treatment approaches. Research has shown that phage display can generate antibodies against *S. aureus* and associated virulence factors, inhibitors that reduce pathogenicity, and ligands that facilitate the targeted release of antibiotics.

### 5.1 Production of antibodies via phage display

Antibodies specific to *S. aureus* play a pivotal role in the body’s humoral response to *S. aureus* infection.^60^ Antibodies can selectively bind and reduce bacterial pathogenicity through various mechanisms, including neutralization of toxins, promotion of phagocytosis, antibody‐dependent cellular cytotoxicity, and activation of the complement system.^60^ Though effective, the synthesis of full antibodies is a time‐consuming, expensive, and technically demanding process.^61^ Monoclonal antibodies are also immunogenic and can induce hypersensitivity reactions.^61^ Fortunately, phage display can be used to overcome some of the limitations of traditional antibody development methods by selecting for highly specific molecules against *S. aureus*.

**FIGURE 6:**
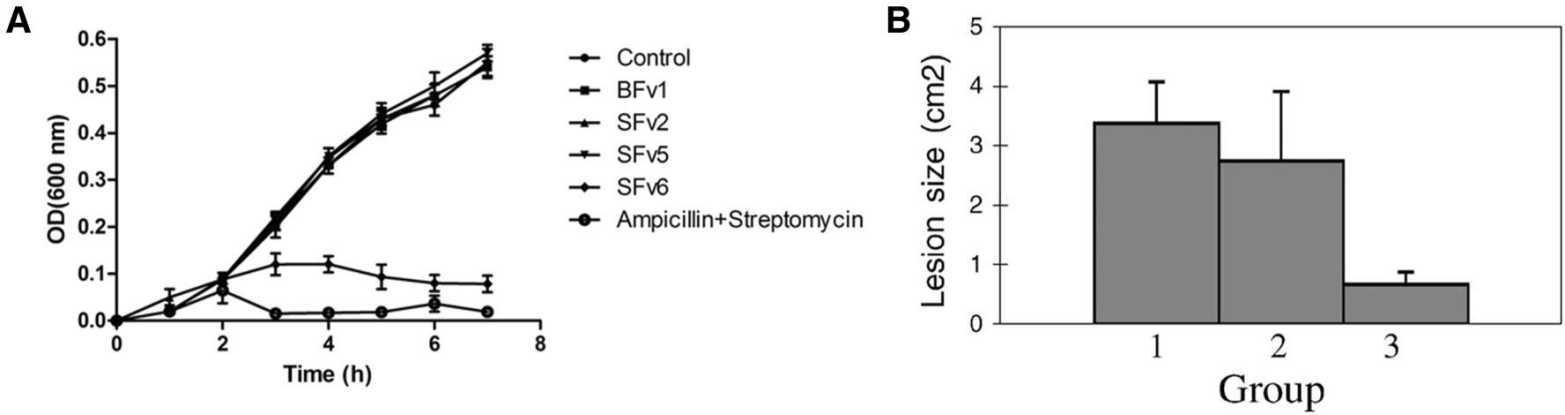
Disinfection of *Staphylococcus aureus* using phage‐displayed peptides. (A) Activity of proteins against *S. aureus* in vitro. Adapted with permission.^61^ Copyright 2016, Elsevier. (B) RBP15 inhibits cellulitis (tested on the murine cutaneous *S. aureus* infection model). Bacteria were treated with: Group 1, PBS control; Group 2, nonrelated GST‐fusion; Group 3, GST‐fusion of RBP15. Adapted with permission.^81^ Copyright 2003, Elsevier.

Single‐chain variable fragments (scFvs) are antibodies that consist of the variable heavy chain and variable light chain connected via a polypeptide linker.^61^ Previous studies have demonstrated their effectiveness in neutralizing bacterial pathogens, thereby reducing pathogenesis.^80^ Li et al. were able to select for a specific scFv against *S. aureus* through phage antibody library technology, which involved selecting for antibodies displayed on the surfaces of phages through phage display (Figure 6A).^61^ To construct the phage display library, hens were immunized with killed *S. aureus* and IgY from their spleen and blood was obtained. The RNA was obtained from the blood and spleen and subsequently reverse transcribed into cDNA. The V_H_ and V_L_ genes were amplified and linked via an amino acid linker to construct the scFv gene repertoire. Phage display of this gene repertoire yielded six blood phages and nine spleen phages. After induced expression, four soluble proteins were obtained, of which one, SFV6, was specific and effective against *S. aureus* infection. Though this study synthesized scFv effective against *S. aureus* in vitro, scFvs are generally unstable and lack the fragment crystallizable (Fc) regions and their associated properties.^60^ To avoid these potential deficiencies, researchers fused the scFv with Fc, resulting in increased stability. These fusion scFv maintained the Fc function of antibodies, which allowed for their prolonged survival and effectiveness in vivo.^60^ Thus, phage display can be used to synthesize scFvs against *S. aureus* that successfully reduce pathogenicity.

Alternatively, antibodies have also been generated that specifically target the toxins characteristic of *S. aureus* infection.^82^ These toxins are implicated in attacking the host defense barriers, and in the case of SEB, help it evade the immune response.^62^ Phage display can be used to screen for peptides that are effectively able to bind to enterotoxins produced by *S. aureus*,^48^, ^63^ such as SEB, a pyrogenic toxin that is implicated in food poisoning in mammals.^83^ Zanganeh et al. utilized phage display in the process of synthesizing nanobodies to specifically select for SEB.^48^ Nanobody genes were obtained from SEB‐immunized camels and were cloned into a vector to obtain a phage display library. SEB‐specific phages were then selected, and the specific anti‐SEB nanobodies were obtained. Though anti‐SEB nanobodies synthesized through phage display are primarily effective in the detection of *S. aureus*, recent studies have shown that the synthesis of antibodies against exotoxins may effectively combat infection.^64^ As diagnosis and administration of antibacterial treatments against *S. aureus* occur after infection, sufficient time has been provided to allow for the synthesis of virulence factors that are implicated in clinical manifestations. However, antibacterial treatments tend to be ineffective against already formed exotoxins.^64^ Utilizing this technique to synthesize antibodies that effectively neutralize virulence factors could significantly reduce the symptoms associated with infection. Thus far, prophylactic and passive therapy treatments of mAbs against SEB have been successful in binding SEB in vivo and reducing virulence.^65^ More research needs to be conducted to substantiate the effectiveness of anti‐SEB nanobodies and elucidate the effectiveness of phage display‐derived antibodies.

Another superantigen synthesized by *S. aureus* is the toxic shock syndrome toxin‐1 (TSST‐1). TSST‐1 is involved in producing large quantities of pro‐inflammatory cytokines that are released after TSST‐1 stimulates T lymphocyte proliferation.^66^ A study conducted by Rukkawattanakul et al. demonstrated the effectiveness of scFv synthesized against TSST‐1 as a treatment against TSS.^67^ scFvs specific to TSST‐1 were synthesized through the use of a phage display library prepared using mRNA from peripheral T lymphocytes of humans. The obtained sequences were used to synthesize scFvs that were used to generate the phage display library. Three clones were effective in directly binding to TSST‐1 and also exhibited complete human scFv sequences. The HuscFvs generated through this approach were effective in preventing mitogenicity and pyrogenicity of TSST‐1. The scFvs were able bind to and neutralize the epitope of TSST‐1, reducing T lymphocyte activation. This subsequently reduced the production of inflammatory cytokines, effectively preventing TSS.^67^ It is evident that using the phage display technique can generate robust antibodies specific for their targeted antigens; however, little is known on duration of the sustained humoral response. Future studies should evaluate the duration and sustained effectives of protection from antibodies derived from phage display libraries.

### 5.2 Production of inhibitors via phage display

The expression of various surface or secretory molecules is responsible for the pathogenicity of *S. aureus.* These virulence factors include molecules that aid colonization of host tissues, toxins involved in lysis of cell membranes, compounds that prevent phagocytosis or improve survival within phagocytes, immunological disguises, superantigens, and antimicrobial resistance proteins.^84^ Given the plethora of virulence factors, phage display libraries can used to screen and identify peptides that are able to inhibit expression and treat infection.

*S. aureus* synthesizes several virulence factors, which are tightly regulated by quorum sensing mechanisms.^83^ *S. aureus* produces a protein referred to as RNAIII activating protein (RAP). RAP activates RNAIII, an enzyme involved in the synthesis of these virulence factors.^83^ Yang et al. used phage display to select for peptides that would selectively bind and inhibit RNAIII, thereby reducing the production of virulence factors (Figure 6B).^81^ Phage display was conducted to select for RAP binding peptides (RBPs) that would inhibit RAP. Two clones were obtained, RBP7 and RBP15, which selectively bound to RAP with high affinity, though RBP15 bound *S. aureus* with higher affinity.^81^ Through northern blotting, they observed that the synthesis of RNAIII was greatly reduced in vitro when utilizing RBP15. In a murine cutaneous model, it was observed that RBP15 was also able to reduce the lesion size in mice implicated with cellulitis. This treatment, however, was only observed to be effective when *S. aureus* was pre‐cultured with RBP15, highlighting a limitation in its translatability. Further research needs to be conducted to assess its effectiveness in vivo and as a treatment strategy for *S. aureus* infection. Additionally, future research should evaluate its effectiveness in different types of *S. aureus* infections.

Currently, the most common treatments used against *S. aureus* are antibiotics consisting of a lactam. Beta‐lactam antibiotics hinder the formation of the cell wall in bacteria by disrupting peptidoglycan layer formation.^85^ These drugs contain a 3‐carbon and one nitrogen ring, referred to as the beta‐lactam ring, which is central to their mechanism of action. Resistances against these lactam antibiotics are rendering them increasingly ineffective against *S. aureus*. A common mechanism believed to confer this antibiotic resistance is the synthesis of beta‐lactamase,^86^ enzymes which hydrolyze and subsequently inactivate the beta‐lactam ring. A new class of proteins referred to as beta‐lactamase inhibitory proteins (BLIPs) effectively inactivate serine beta‐lactamases. Phage display can be utilized to explore treatment strategies involving BLIPs by identifying peptides involved in binding and inhibiting beta‐lactamases (i.e., identifying BLIPs).^87^ In a study conducted by Huang et al., phage display and SPOT synthesis were used to select for a peptide that bound directly to and inhibited TEM‐1, a class A beta‐lactamase.^87^ This approach yielded peptides that affected the pathogenicity of *S. aureus* by directly inhibiting the molecules that confer resistance against antimicrobial treatments. Alternatively, phage display can also be used to produce phages that facilitate the direct targeting of beta‐lactamases by BLIPs.^88^ Researchers used phage display to express a BLIP‐g3p fusion protein on the surface of an M13 phage, which would alter the specificity of BLIP. This strategy could be exploited to understand the important residues involved in the binding of BLIP to beta‐lactamase and identify the different variants that bind to and inhibit beta‐lactamases.

### 5.3 Production of drug carriers via phage display

One such method to increase efficacy of existing medications is by delivering antibiotics selectively to the infection site, thereby increasing local potency and reducing the risk of adverse effects.^89^, ^90^ Active targeting through ligand‐functionalized drug carriers has already shown to increase the therapeutic index of existing antibiotics.^91^, ^92^ While previous research has recognized phage display as a powerful screening tool for finding ligands that bind to tumors^93^ or injured tissues,^94^ phage display has demonstrated promise as a tool capable of screening for peptides that can selectively home to *S. aureus* to combat infection.

Yacoby et al. displayed the effectiveness and potential of using bacteriophages as loaded drug carriers against *S. aureus*.^95^ The study’s aim was threefold: to increase potency by facilitating a high concentration of the drug in a microenvironment around the target cell, reduce general toxicity of the drugs, and reintroduce these nonspecific toxic substances as therapeutic options against drug‐resistant bacteria. Chloramphenicol, a potent antibiotic, was conjugated to M13 phages via a linker. The drug is inactive when conjugated to the phage, reducing its toxicity within the body. Only after the bond is cleaved via a serum esterase, the drug can elicit its bacteriostatic effects. The researchers observed that this strategy was effective in enabling the controlled release of the drug. The selective targeting and binding of *S. aureus* were achieved through two strategies: phage‐displayed peptides specific to *S. aureus* and antibody‐mediated targeting using IgG linked to the phage. Yacoby et al. demonstrated that phage binding would release chloramphenicol, thereby inhibiting *S. aureus* growth. There was one potential limitation to their approach. Chloramphenicol is a hydrophobic drug, which reduced the overall loading capacity of this drug to the phage.^95^ In a further study, they enhanced the targeting using antibodies in which the aminoglycoside neomycin was used as a solubility‐enhancing linker.^96^ The loading capacity increased to over 40,000 drug molecules per phage, proving effective in reducing *S. aureus* growth in vitro.

Drug‐loaded nanoparticles are an emerging approach that enhances the pharmacokinetics of antibiotics. Conjugated ligands selected through phage display can increase the affinity of this drug‐carrier system for the target tissue. Phage display can be utilized to detect specific peptides that can bind to *S. aureus.* These peptides are incorporated into the nanoparticle drug delivery systems that improve antibiotic delivery and bioavailability.^89^ Hussain et al., using phage display and in vivo screening to identify peptides that bind to *S. aureus*, were able identify a cyclic nine amino acid peptide CARG that increased the delivery of vancomycin.^89^ The peptide was conjugated to porous silicon nanoparticles and intravenously injected into mice challenged with *S. aureus*. This strategy was observed to effectively reduce the toxicity of vancomycin while increasing the likelihood of survival from bacterial lung infection in comparison to free vancomycin treatment. As a result of this systems’ ability to facilitate the accumulation of nanoparticles in a targeted manner, it has been effective in reducing the overall dose required for treatment. Limitations in this method arise from the site of delivery. Future studies should evaluate how broadly applicable this mode of delivery is to treating various human conditions.

## 6 PREVENTION OF *S. AUREUS* USING PHAGE DISPLAY

**FIGURE 7:**
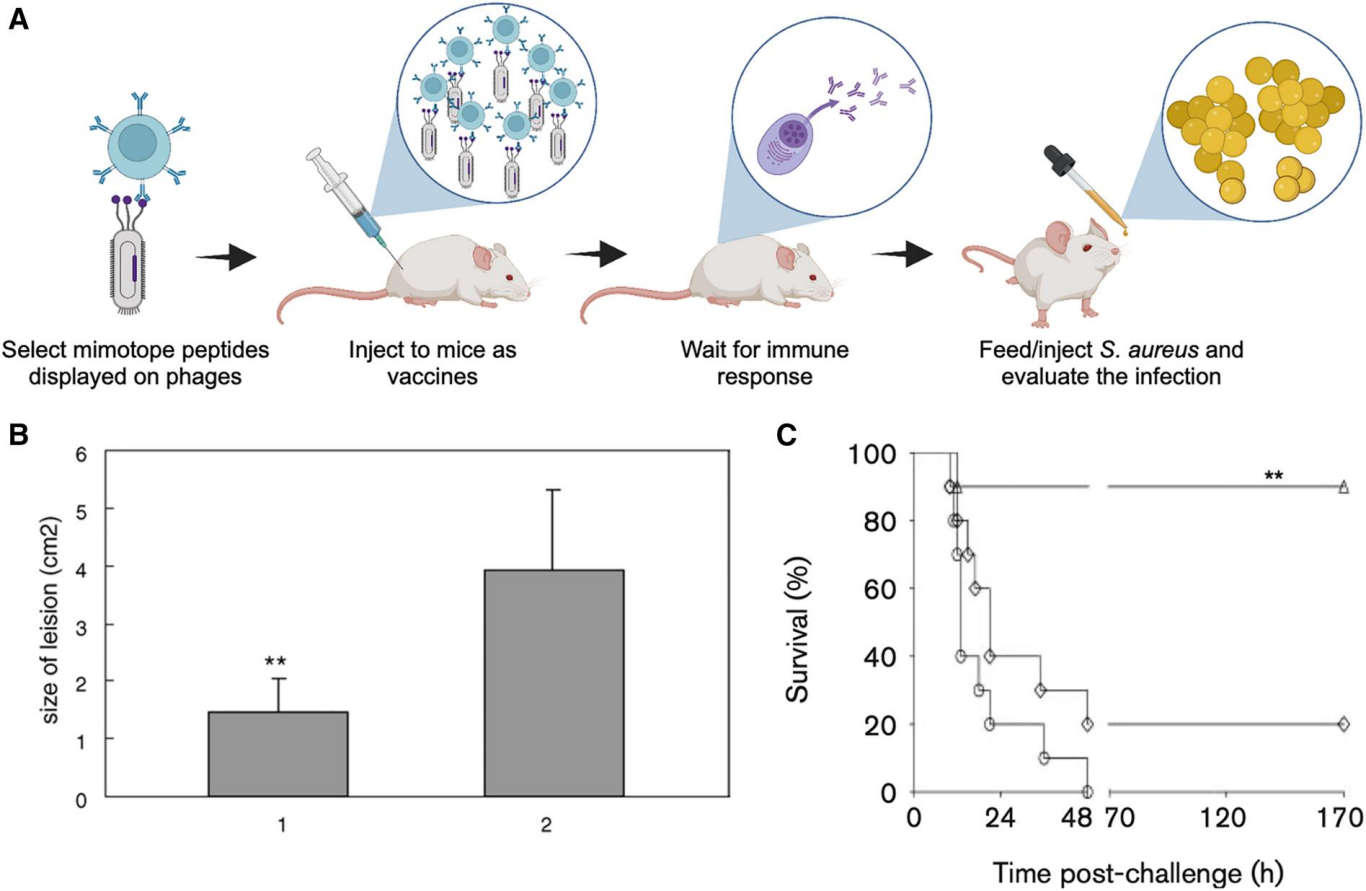
Prevention of *Staphylococcus aureus* using phage‐displayed peptides. (A) Schematic of using and evaluating phage‐displayed mimotopes as vaccines, created with BioRender.com. (B) Mice immunized with mimotopes are protected from *S. aureus* infection. Group 1: Mice immunized with R13; Group 2: Control mice immunized with no fusion peptide. **p < 0.001. Adapted with permission.^97^ Copyright 2003, Elsevier. (C) Mimotope vaccines enhance the clearance of bacteria and survival of immunized mice challenged with *S. aureus*. Adapted with permission.^51^ Copyright 2011, Microbiology Society.

In recent years, phage display has been utilized to select peptides that can be inoculated in patients to induce immunity against *S. aureus* (Figure 7A).^98^ One approach involves targeting the quorum sensing pathway of *S. aureus.* In *S. aureus*, the accessory gene regulator (*agr*) quorum‐sensing mechanism is implicated producing virulence factors including toxic shock syndrome toxin‐1 and hemolysins and is critical for pathogenesis.^99^ This quorum‐sensing pathway regulates gene expression in a density‐dependent manner and is pivotal for cell communication. The *agr* locus transcripts are RNAII and RNAIII, of which RNAIII encodes for and increases the expression of several virulence factors. RBPs mechanistically disrupt cell‐cell communication by selectively binding the signaling molecule RAP, thereby inhibiting the synthesis of the virulence factors produced by RNAIII and reducing virulence. Yang et al. generated antibodies against RAP through immunizing rabbits.^97^ After the production and purification of anti‐RAP antibodies, they identified the mimotope of RAP using a 12‐mer phage display library to select for peptides that would selectively bind the anti‐RAP antibodies. Among these peptides, R13 was determined to be effective in preventing *S. aureus* infection in mice when administered prophylactically, providing protection even 6 months after immunization. Through effectively mimicking RAP, a protective immune response was induced, demonstrating the potential for phage display as an effective method for selecting and generating peptide vaccines to prevent *S. aureus* infection (Figure 7B).^97^

Vaccines can also be used to generate immunity against different recognizable components of the *S. aureus* pathogen. Despite peptidoglycan being a common and highly conserved component of gram‐positive bacteria, such as *S. aureus*, it is a thymus‐independent antigen that is insufficiently immunogenic and fails to induce a sufficient memory B cell response.^51^ To increase immunogenicity, Chen et al. used phage display to select for peptide mimics of peptidoglycan that could act as thymus‐dependent antigens.^51^ These peptides selectively targeted anti‐PGN mAbs, induced an antibody response to PGN and other cell‐wall fragments, and improved survival rates and bacterial clearance of immunized mice (Figure 7C). While existing *S. aureus* vaccine candidates exhibit limitations in their ability to induce protective immunity,^100^, ^101^ generating peptide mimotopes from phage display represents a new potential approach in vaccine development against *S*. *aureus.* Future studies must demonstrate robust and sustained protection.

## 7 CONCLUSION

The research area of infectious diseases continues to expand. While techniques for detection and treatments strategies, such as ELISA and antibiotics, respectively, proved initially effective at mitigating the emerging risk of *S. aureus*, they have since become insufficient due to the adaptations made by the bacterium. Phage display represents a dual‐purpose platform technology to provide better detection of *S. aureus* and mitigate its healthcare burden. It is phage display’s intrinsic adaptive capability to quickly identify selective proteins for further optimization that makes it such a versatile solution for *S. aureus* infections. In addition, phages have been used as building blocks for constructing functional biomaterials, such as bulk hydrogels as loading scaffolds^102^ and antimicrobial materials,^28^, ^103^ spheres as cell‐culture substrates,^104^ and 2D films as sensors.^105^, ^106^ Combining the phage display we discussed in this review with these functional biomaterials would potentially bring more powerful and comprehensive strategies to treat pathogens like *S. aureus*. To this end, phage display has already yielded important results in studying *S. aureus* infections. It has developed reliable detection methods and serves as a useful tool for epitope mapping to identify novel drug targets and vaccine candidates. Given its near unlimited design potential, phage display will continue to play an important role in studying infectious diseases, specifically *S. aureus* infections, so that we may alleviate its toll on our public health systems.

## AUTHOR CONTRIBUTIONS

Lei Tian and Kyle Jackson prepared the figures and contributed to writing of the manuscript. Michael Chan, Ahmed Saif, and Leon He made important contributions to writing the manuscript. Lei Tian, Tohid F. Didar, and Zeinab Hosseinidoust conceptualized and supervised the project and guided the data analysis and manuscript writing.

## CONFLICT OF INTEREST

Authors declare no competing interest.

## References

[1] F. R. DeLeo , M. Otto , B. N. Kreiswirth , H. F. Chambers , Lancet 2010, 375, 1557. 20206987 10.1016/S0140-6736(09)61999-1 PMC3511788

[2] T. Yamamoto , A. Nishiyama , T. Takano , S. Yabe , W. Higuchi , O. Razvina , D. Shi , J. Infect. Chemother. 2010, 16, 225. 20336341 10.1007/s10156-010-0045-9 PMC7088255

[3] E. O. Irek , A. A. Amupitan , T. O. Obadare , A. O. Aboderin , Afr. J. Lab. Med. 2018, 7, a796. 10.4102/ajlm.v7i2.796 PMC6296034 30568902

[4] S. Y. C. Tong , J. S. Davis , E. Eichenberger , T. L. Holland , V. G. Fowler , Clin. Microbiol. Rev. 2015, 28, 603. 26016486 10.1128/CMR.00134-14 PMC4451395

[5] V. J. Torres , A. S. Attia , W. J. Mason , M. I. Hood , B. D. Corbin , F. C. Beasley , K. L. Anderson , D. L. Stauff , W. H. McDonald , L. J. Zimmerman , D. B. Friedman , D. E. Heinrichs , P. M. Dunman , E. P. Skaar , Infect. Immun. 2010, 78, 1618. 20100857 10.1128/IAI.01423-09 PMC2849423

[6] G. Y. C. Cheung , R. Wang , B. A. Khan , D. E. Sturdevant , M. Otto , Infect. Immun. 2011, 79, 1927. 21402769 10.1128/IAI.00046-11 PMC3088142

[7] F. Lamret , J. Varin‐Simon , F. Velard , C. Terryn , C. Mongaret , M. Colin , S. C. Gangloff , F. Reffuveille , Front. Microbiol. 2021, 12, 714994. 34557170 10.3389/fmicb.2021.714994 PMC8453086

[8] M. M. Dinges , P. M. Orwin , P. M. Schlievert , Clin. Microbiol. Rev. 2000, 13, 16. 10627489 10.1128/cmr.13.1.16-34.2000 PMC88931

[9] C. Kong , H. M. Neoh , S. Nathan , Toxins 2016, 8, 72. 26999200 10.3390/toxins8030072 PMC4810217

[10] M. Jusko , J. Potempa , T. Kantyka , E. Bielecka , H. K. Miller , M. Kalinska , G. Dubin , P. Garred , L. N. Shaw , A. M. Blom , J. Innate Immun. 2014, 6, 31. 23838186 10.1159/000351458 PMC3972074

[11] M. McAdow , A. C. DeDent , C. Emolo , A. G. Cheng , B. N. Kreiswirth , D. M. Missiakas , O. Schneewind , Infect. Immun. 2012, 80, 3389. 22825443 10.1128/IAI.00562-12 PMC3457572

[12] K. Tam , V. J. Torres , Microbiol. Spectr. 2019, 7, 16. 10.1128/microbiolspec.gpp3-0039-2018 PMC6422052 30873936

[13] M. Idrees , S. Sawant , N. Karodia , A. Rahman , Int. J. Environ. Res. Public Health 2021, 18, 7602. 34300053 10.3390/ijerph18147602 PMC8304105

[14] J. S. Nanra , S. M. Buitrago , S. Crawford , J. Ng , P. S. Fink , J. Hawkins , I. L. Scully , L. K. McNeil , J. M. Aste‐Amezaga , D. Cooper , K. U. Jansen , A. S. Anderson , Hum. Vaccines Immunother. 2013, 9, 480. 10.4161/hv.23223 PMC3891703 23249887

[15] V. Thammavongsa , H. K. Kim , D. Missiakas , O. Schneewind , Nat. Rev. Microbiol. 2015, 13, 529. 26272408 10.1038/nrmicro3521 PMC4625792

[16] F. R. DeLeo , B. A. Diep , M. Otto , Infect. Dis. Clin. North Am. 2009, 23, 17. 19135914 10.1016/j.idc.2008.10.003 PMC2748223

[17] T. J. Foster , Nat. Rev. Microbiol. 2005, 3, 948. 16322743 10.1038/nrmicro1289

[18] E. J. G. Pollitt , P. T. Szkuta , N. Burns , S. J. Foster , PLOS Pathog 2018, 14, e1007112. 29902272 10.1371/journal.ppat.1007112 PMC6019756

[19] Y. Zheng , L. He , T. K. Asiamah , M. Otto , Environ. Microbiol. 2018, 20, 3141. 29633455 10.1111/1462-2920.14129 PMC6162163

[20] G. Pietrocola , D. Campoccia , C. Motta , L. Montanaro , C. R. Arciola , P. Speziale , Int. J. Mol. Sci. 2022, 23, 5958. 35682632 10.3390/ijms23115958 PMC9180976

[21] A. Papkou , J. Hedge , N. Kapel , B. Young , R. C. MacLean , Nat. Commun. 2020, 11, 3970. 32769975 10.1038/s41467-020-17735-y PMC7414891

[22] T. J. Foster , FEMS Microbiol. Rev. 2017, 41, 430. 28419231 10.1093/femsre/fux007

[23] A. S. Lee , H. de Lencastre , J. Garau , J. Kluytmans , S. Malhotra‐Kumar , A. Peschel , S. Harbarth , Nat. Rev. Dis. Prim. 2018, 4, 1. 29849094 10.1038/nrdp.2018.33

[24] World Health Organization, https://www.who.int/news/item/27‐02‐2017‐who‐publishes‐list‐of‐bacteria‐for‐which‐new‐antibiotics‐are‐urgently‐needed (Accessed: 27th August 2022).

[25] C. J. L. Murray , K. S. Ikuta , F. Sharara , L. Swetschinski , G. Robles Aguilar , A. Gray , C. Han , C. Bisignano , P. Rao , E. Wool , S. C. Johnson , A. J. Browne , M. G. Chipeta , F. Fell , S. Hackett , G. Haines‐Woodhouse , B. H. Kashef Hamadani , E. A. P. Kumaran , B. McManigal , R. Agarwal , S. Akech , S. Albertson , J. Amuasi , J. Andrews , A. Aravkin , E. Ashley , F. Bailey , S. Baker , B. Basnyat , A. Bekker , R. Bender , A. Bethou , J. Bielicki , S. Boonkasidecha , J. Bukosia , C. Carvalheiro , C. Castañeda‐Orjuela , V. Chansamouth , S. Chaurasia , S. Chiurchiù , F. Chowdhury , A. J. Cook , B. Cooper , T. R. Cressey , E. Criollo‐Mora , M. Cunningham , S. Darboe , N. P. J. Day , M. De Luca , K. Dokova , A. Dramowski , S. J. Dunachie , T. Eckmanns , D. Eibach , A. Emami , N. Feasey , N. Fisher‐Pearson , K. Forrest , D. Garrett , P. Gastmeier , A. Z. Giref , R. C. Greer , V. Gupta , S. Haller , A. Haselbeck , S. I. Hay , M. Holm , S. Hopkins , K. C. Iregbu , J. Jacobs , D. Jarovsky , F. Javanmardi , M. Khorana , N. Kissoon , E. Kobeissi , T. Kostyanev , F. Krapp , R. Krumkamp , A. Kumar , H. H. Kyu , C. Lim , D. Limmathurotsakul, M. J. Loftus, M. Lunn, J. Ma, N. Mturi, T. Munera‐Huertas, P. Musicha, M. M. Mussi‐Pinhata, T. Nakamura, R. Nanavati, S. Nangia, P. Newton, C. Ngoun, A. Novotney, D. Nwakanma, C. W. Obiero, A. Olivas‐Martinez, P. Olliaro, E. Ooko, E. Ortiz‐Brizuela, A. Y. Peleg, C. Perrone, N. Plakkal, A. Ponce‐de‐Leon, M. Raad, T. Ramdin, A. Riddell, T. Roberts, J. V. Robotham, A. Roca, K. E. Rudd, N. Russell, J. Schnall, J. A. G. Scott, M. Shivamallappa, J. Sifuentes‐Osornio, N. Steenkeste, A. J. Stewardson, T. Stoeva, N. Tasak, A. Thaiprakong, G. Thwaites, C. Turner, P. Turner, H. R. van Doorn, S. Velaphi, A. Vongpradith, H. Vu, T. Walsh, S. Waner, T. Wangrangsimakul, T. Wozniak, P. Zheng, B. Sartorius, A. D. Lopez, A. Stergachis, C. Moore, C. Dolecek, M. Naghavi, Lancet 2022, 399, 629.35065702

[26] World Health Organization , Antibacterial Agents in Clinical Development: An Analysis of the Antibacterial Clinical Development Pipeline, World Health Organization , Geneva 2019.

[27] L. Tian , K. Jackson , A. Zhang , Z. Wan , A. Saif , Z. Hosseinidoust , Can. J. Chem. Eng. 2022, 100, 2191.

[28] A. Peivandi , L. Tian , R. Mahabir , Z. Hosseinidoust , Chem. Mater. 2019, 31, 5442.

[29] K. Jackson , A. Peivandi , M. Fogal , L. Tian , Z. Hosseinidoust , ACS Appl. Bio Mater. 2021, 4, 2262. 10.1021/acsabm.0c01557 35014350

[30] A. Peivandi , K. Jackson , L. Tian , L. He , A. Mahmood , C. Fradin , Z. Hosseinidoust , ACS Biomater. Sci. Eng. 2022, 8, 340. 34905337 10.1021/acsbiomaterials.1c01112

[31] S. Mimmi , D. Maisano , I. Quinto , E. Iaccino , Trends Pharmacol. Sci. 2019, 40, 87. 30606501 10.1016/j.tips.2018.12.005

[32] G. P. Smith , V. A. Petrenko , Chem. Rev. 1997, 97, 391. 11848876 10.1021/cr960065d

[33] J. Pande , M. M. Szewczyk , A. K. Grover , Biotechnol. Adv. 2010, 28, 849. 20659548 10.1016/j.biotechadv.2010.07.004

[34] M. S. Newton , Y. Cabezas‐Perusse , C. L. Tong , B. Seelig , ACS Synth. Biol. 2020, 9, 181. 31891492 10.1021/acssynbio.9b00419 PMC8203280

[35] J. Bazan , I. Całkosiński , A. Gamian , Hum. Vaccin. Immunother. 2012, 8, 1817. 22906939 10.4161/hv.21703 PMC3656071

[36] G. P. Smith , Science 1985, 228, 1315. 4001944 10.1126/science.4001944

[37] S. F. Parmley , G. P. Smith , Gene 1988, 73, 305. 3149606 10.1016/0378-1119(88)90495-7

[38] H. Wang , Y. Chang , C. Hu , C. Kao , Y. Yu , S. Lim , K. Mou , ACS Synth. Biol. 2021, 10, 2087. 34342970 10.1021/acssynbio.1c00266

[39] J. Zhu , N. Ananthaswamy , S. Jain , H. Batra , W. Tang , D. A. Lewry , M. L. Richards , S. A. David , P. B. Kilgore , J. Sha , A. Drelich , C. T. K. Tseng , A. K. Chopra , V. B. Rao , Sci. Adv. 2021, 7, eabh1547. 34516878 10.1126/sciadv.abh1547 PMC8442874

[40] J. K. Liu , D. Lubelski , D. L. Schonberg , Q. Wu , J. S. Hale , W. A. Flavahan , E. E. Mulkearns‐Hubert , J. Man , A. B. Hjelmeland , J. Yu , J. D. Lathia , J. N. Rich , Cell Death Differ. 2014, 21, 1325. 24832468 10.1038/cdd.2014.65 PMC4085538

[41] Q. Chen , L. Zhang , Y. Feng , F. Shi , Y. Wang , P. Wang , L. Liu , J. Mater. Chem. B 2018, 6, 7643. 32254886 10.1039/c8tb01835a

[42] L. M. De Plano , S. Scibilia , M. G. Rizzo , D. Franco , A. M. Mezzasalma , S. P. P. Guglielmino , Appl. Phys. A 2018, 124, 787.

[43] L. M. De Plano , E. Fazio , M. G. Rizzo , D. Franco , S. Carnazza , S. Trusso , F. Neri , S. P. Guglielmino , J. Immunol. Methods 2019, 465, 45. 30552870 10.1016/j.jim.2018.12.004

[44] J. Xiong , W. Wang , Z. Fu , Microchim. Acta 2017, 184, 4197.

[45] L. M. De Plano , S. Carnazza , G. M. Messina , M. G. Rizzo , G. Marletta , S. P. Guglielmino , Colloids Surf. B Biointerfaces 2017, 157, 473. 28654884 10.1016/j.colsurfb.2017.05.081

[46] S. S. Rao , K. V. K. Mohan , Y. Gao , C. D. Atreya , Microbiol. Res. 2013, 168, 106. 23017232 10.1016/j.micres.2012.07.004

[47] P. Liu , L. Han , F. Wang , V. A. Petrenko , A. Liu , Biosens. Bioelectron. 2016, 82, 195. 27085951 10.1016/j.bios.2016.03.075

[48] S. Zanganeh , H. Rouhani Nejad , J. F. Mehrabadi , R. Hosseini , B. Shahi , Z. Tavassoli , A. Aramvash , Appl. Biochem. Biotechnol. 2018, 187, 493. 29984392 10.1007/s12010-018-2762-y

[49] E. A. Soykut , F. C. Dudak , İ. H. Boyacı , Biochem. Biophys. Res. Commun. 2008, 370, 104. 18359289 10.1016/j.bbrc.2008.03.065 PMC7117543

[50] E. R. Goldman , M. P. Pazirandeh , J. M. Mauro , K. D. King , J. C. Frey , G. P. Anderson , J. Mol. Recognit. 2000, 13, 382. 11114071 10.1002/1099-1352(200011/12)13:6<382::AID-JMR511>3.0.CO;2-W

[51] Y. Chen , B. Liu , D. Yang , X. Li , L. Wen , P. Zhu , N. Fu , J. Med. Microbiol. 2011, 60, 995. 21436375 10.1099/jmm.0.028647-0

[52] G. Yang , Y. Gao , J. Dong , C. Liu , Y. Xue , M. Fan , B. Shen , N. Shao , J. Biol. Chem. 2005, 280, 27431. 15908434 10.1074/jbc.M501127200

[53] R. R. Graef , G. P. Anderson , K. A. Doyle , D. Zabetakis , F. N. Sutton , J. L. Liu , J. Serrano‐Gonzalez , E. R. Goldman , L. A. Cooper , BMC Biotechnol 2011, 11, 86. 21933444 10.1186/1472-6750-11-86 PMC3193169

[54] E. Gustafsson , P. J. Haas , B. Walse , M. Hijnen , C. Furebring , M. Ohlin , J. A. van Strijp , K. P. van Kessel , BMC Immunol 2009, 10, 13. 19284584 10.1186/1471-2172-10-13 PMC2662796

[55] C. Fevre , J. Bestebroer , M. M. Mebius , C. J. C. de Haas , J. A. G. van Strijp , J. R. Fitzgerald , P. J. A. Haas , Cell. Microbiol. 2014, 16, 1646. 24840181 10.1111/cmi.12313

[56] Y. Li , Y. Liu , Z. Li , M. Liu , L. Liu , X. Wang , X. Wang , J. Suo , W. Han , Res. Vet. Sci. 2013, 94, 490. 23178048 10.1016/j.rvsc.2012.10.013

[57] M. Wang , L. Zhai , W. Yu , Y. Wei , L. Wang , S. Liu , W. Li , X. Li , S. Yu , X. Chen , H. Zhang , J. Chen , Z. Feng , L. Yu , Y. Cui , PLoS One 2018, 13, e0190452. 29304128 10.1371/journal.pone.0190452 PMC5755776

[58] J. Ma , Y. Wei , L. Zhang , X. Wang , D. Yao , D. Liu , W. Liu , S. Yu , Y. Yu , Z. Wu , L. Yu , Z. Zhu , Y. Cui , J. Med. Microbiol. 2018, 67, 423. 29458526 10.1099/jmm.0.000633

[59] Y. Gao , J. Dong , X. Zhang , Y. Liu , Q. Lu , J. Feng , X. Tan , G. Yang , Cell Biochem. Biophys. 2013, 66, 753. 23420525 10.1007/s12013-013-9520-0

[60] S. Nian , T. Wu , Y. Ye , X. Wang , W. Xu , Q. Yuan , BMC Immunol. 2016, 17, 1. 27129873 10.1186/s12865-016-0146-z PMC4850644

[61] J. Li , Y. Xu , X. Wang , Y. Li , L. Wang , X. Li , Int. Immunopharmacol. 2016, 35, 149. 27046516 10.1016/j.intimp.2016.02.024

[62] H. Karauzum , G. Chen , L. Abaandou , M. Mahmoudieh , A. R. Boroun , S. Shulenin , V. S. Devi , E. Stavale , K. L. Warfield , L. Zeitlin , C. J. Roy , S. S. Sidhu , M. J. Aman , J. Biol. Chem. 2012, 287, 25203. 22645125 10.1074/jbc.M112.364075 PMC3408135

[63] A. V. Lomonosova , A. G. Laman , K. K. Fursova , A. O. Shepelyakovskaya , Y. V. Vertiev , F. A. Brovko , E. V. Grishin , MAbs 2011, 3, 513. 22123058 10.4161/mabs.3.6.18089 PMC3242837

[64] M. J. Karau , M. E. Tilahun , A. Krogman , B. A. Osborne , R. A. Goldsby , C. S. David , J. N. Mandrekar , R. Patel , G. Rajagopalan , Virulence 2017, 8, 1148. 27925510 10.1080/21505594.2016.1267894 PMC5711449

[65] A. K. Varshney , X. Wang , M. D. Scharff , J. MacIntyre , R. S. Zollner , O. V. Kovalenko , L. R. Martinez , F. R. Byrne , B. C. Fries , J. Infect. Dis. 2013, 208, 2058. 23922375 10.1093/infdis/jit421 PMC3836467

[66] P. Speziale , S. Rindi , G. Pietrocola , Microorganisms 2018, 6, 25. 29533985 10.3390/microorganisms6010025 PMC5874639

[67] T. Rukkawattanakul , N. Sookrung , W. Seesuay , N. Onlamoon , P. Diraphat , W. Chaicumpa , N. Indrawattana , Toxins 2017, 9, 50. 28218671 10.3390/toxins9020050 PMC5331430

[68] R. M. Braga , M. N. Dourado , W. L. Araújo , Brazilian J. Microbiol. 2016, 47, 86. 10.1016/j.bjm.2016.10.005 PMC5156507 27825606

[69] Y. Oogai , M. Matsuo , M. Hashimoto , F. Kato , M. Sugai , H. Komatsuzawa , Appl. Environ. Microbiol. 2011, 77, 8097. 21926198 10.1128/AEM.05316-11 PMC3208999

[70] M. Otto , Curr. Opin. Microbiol. 2014, 17, 32. 24581690 10.1016/j.mib.2013.11.004 PMC3942668

[71] I. V. Pinchuk , E. J. Beswick , V. E. Reyes , Toxins 2010, 2, 2177. 22069679 10.3390/toxins2082177 PMC3153290

[72] S. Wu , N. Duan , H. Gu , L. Hao , H. Ye , W. Gong , Z. Wang , Toxins 2016, 8, 176. 27348003 10.3390/toxins8070176 PMC4963824

[73] J. L. Liu , E. R. Goldman , D. Zabetakis , S. A. Walper , K. B. Turner , L. C. Shriver‐Lake , G. P. Anderson , Microb. Cell Fact. 2015, 14, 158. 26449768 10.1186/s12934-015-0340-3 PMC4599338

[74] S. S. Agasti , S. Rana , M. H. Park , C. K. Kim , C. You , V. M. Rotello , Adv. Drug Deliv. Rev. 2010, 62, 316. 19913581 10.1016/j.addr.2009.11.004 PMC2827652

[75] J. Huang , A. Gutteridge , W. Honda , M. Kanehisa , BMC Bioinform. 2006, 7, 451. 10.1186/1471-2105-7-451 PMC1618411 17038191

[76] J. Luzar , B. Štrukelj , M. Lunder , Allergy Eur. J. Allergy Clin. Immunol. 2016, 71, 1526. 10.1111/all.12965 27341497

[77] A. Luzzago , F. Felici , A. Tramontano , A. Pessi , R. Cortese , Gene 1993, 128, 51. 7685301 10.1016/0378-1119(93)90152-s

[78] A. J. Pollard , E. M. Bijker , Nat. Rev. Immunol. 2021, 21, 83. 33353987 10.1038/s41577-020-00479-7 PMC7754704

[79] F. Bagnoli , R. Rappuoli , G. Grandi , Staphylococcus aureus, Current Topics in Microbiology and Immunology, Vol. 409, Springer International Publishing AG, Cham 2017.

[80] S. I. Sapats , H. G. Heine , L. Trinidad , G. J. Gould , A. J. Foord , S. G. Doolan , S. Prowse , J. Ignjatovic , Arch. Virol. 2003, 148, 497. 12607101 10.1007/s00705-002-0931-2

[81] G. Yang , H. Cheng , C. Liu , Y. Xue , Y. Gao , N. Liu , B. Gao , D. Wang , S. Li , B. Shen , N. Shao , Peptides 2003, 24, 1823. 15019215 10.1016/j.peptides.2003.09.017

[82] R. Wang , S. Fang , D. Wu , J. Lian , J. Fan , Y. Zhang , S. Wang , W. Lin , Appl. Environ. Microbiol. 2012, 78, 4967. 22562997 10.1128/AEM.00435-12 PMC3416367

[83] K. Hodyra , K. Dąbrowska , Arch. Immunol. Ther. Exp. 2015, 63, 117. 10.1007/s00005-014-0305-y PMC4359349 25048831

[84] M. J. J. B. Sibbald , A. K. Ziebandt , S. Engelmann , M. Hecker , A. de Jong , H. J. M. Harmsen , G. C. Raangs , I. Stokroos , J. P. Arends , J. Y. F. Dubois , J. M. van Dijl , Microbiol. Mol. Biol. Rev. 2006, 70, 755. 16959968 10.1128/MMBR.00008-06 PMC1594592

[85] S. Shaikh , J. Fatima , S. Shakil , S. M. D. Rizvi , M. A. Kamal , Saudi J. Biol. Sci. 2015, 22, 90. 25561890 10.1016/j.sjbs.2014.08.002 PMC4281622

[86] M. E. Ibrahim , M. Abbas , A. M. Al‐Shahrai , B. K. Elamin , Can. J. Infect. Dis. Med. Microbiol. 2019, 2019, 6054694. 31346353 10.1155/2019/6054694 PMC6617866

[87] W. Huang , Z. Beharry , Z. Zhang , T. Palzkill , Protein Eng 2003, 16, 853. 14631075 10.1093/protein/gzg108

[88] W. Huang , J. Petrosino , T. Palzkill , Antimicrob. Agents Chemother. 1998, 42, 2893. 9797222 10.1128/aac.42.11.2893 PMC105962

[89] S. Hussain , J. Joo , J. Kang , B. Kim , G. B. Braun , Z. G. She , D. Kim , A. P. Mann , T. Molder , T. Teesalu , S. Carnazza , S. Guglielmino , M. J. Sailor , E. Ruoslahti , Nat. Biomed. Eng. 2018, 2, 95. 29955439 10.1038/s41551-017-0187-5 PMC6015743

[90] A. Maus , L. Strait , D. Zhu , Eng. Regen. 2021, 2, 31. 38620592 10.1016/j.engreg.2021.03.001 PMC7988306

[91] Y. Yeh , T. Huang , S. Yang , C. Chen , J. Fang , Front. Chem. 2020, 8, 286. 32391321 10.3389/fchem.2020.00286 PMC7193053

[92] Y. Liu , Q. Chen , Y. Sun , L. Chen , Y. Yuan , M. Gu , Eng. Regen. 2021, 2, 206.

[93] K. N. Sugahara , T. Teesalu , P. P. Karmali , V. R. Kotamraju , L. Agemy , O. M. Girard , D. Hanahan , R. F. Mattrey , E. Ruoslahti , Cancer Cell 2009, 16, 510. 19962669 10.1016/j.ccr.2009.10.013 PMC2791543

[94] A. P. Mann , P. Scodeller , S. Hussain , J. Joo , E. Kwon , G. B. Braun , T. Molder , Z. She , V. R. Kotamraju , B. Ranscht , S. Krajewski , T. Teesalu , S. Bhatia , M. J. Sailor , E. Ruoslahti , Nat. Commun. 2016, 7, 11980. 27351915 10.1038/ncomms11980 PMC4931241

[95] I. Yacoby , M. Shamis , H. Bar , D. Shabat , I. Benhar , Antimicrob. Agents Chemother. 2006, 50, 2087. 16723570 10.1128/AAC.00169-06 PMC1479106

[96] I. Yacoby , H. Bar , I. Benhar , Antimicrob. Agents Chemother. 2007, 51, 2156. 17404004 10.1128/AAC.00163-07 PMC1891362

[97] G. Yang , Y. Gao , J. Dong , Y. Xue , M. Fan , B. Shen , C. Liu , N. Shao , Vaccine 2006, 24, 1117. 16359760 10.1016/j.vaccine.2005.09.004

[98] Q. Bao , X. Li , G. Han , Y. Zhu , C. Mao , M. Yang , Adv. Drug Deliv. Rev. 2019, 145, 40. 30594492 10.1016/j.addr.2018.12.013

[99] J. M. Yarwood , P. M. Schlievert , J. Clin. Invest. 2003, 112, 1620. 14660735 10.1172/JCI20442 PMC281656

[100] M. Schmaler , N. J. Jann , F. Ferracin , R. Landmann , J. Immunol. 2011, 186, 443. 21131426 10.4049/jimmunol.1001407

[101] B. Spellberg , R. S. Daum , Hum. Vaccin. 2010, 6, 857. 20930569 10.4161/hv.6.10.12469 PMC3049966

[102] S. M. Jung , J. Qi , D. Oh , A. Belcher , J. Kong , Adv. Funct. Mater. 2017, 27, 1603203.

[103] L. Tian , L. He , K. Jackson , A. Saif , S. Khan , Z. Wan , T. Didar , Z. Hosseinidoust , Nature Communications 2022, 13, 7158. 10.1038/s41467-022-34803-7 PMC9723106 36470891

[104] G. R. Souza , J. R. Molina , R. M. Raphael , M. G. Ozawa , D. J. Stark , C. S. Levin , L. F. Bronk , J. S. Ananta , J. Mandelin , M. M. Georgescu , J. A. Bankson , J. G. Gelovani , T. C. Killian , W. Arap , R. Pasqualini , Nat. Nanotechnol. 2010, 5, 291. 20228788 10.1038/nnano.2010.23 PMC4487889

[105] W. J. Chung , J. W. Oh , K. Kwak , B. Y. Lee , J. Meyer , E. Wang , A. Hexemer , S. W. Lee , Nature 2011, 478, 364. 22012394 10.1038/nature10513

[106] J. W. Oh , W. J. Chung , K. Heo , H. E. Jin , B. Y. Lee , E. Wang , C. Zueger , W. Wong , J. Meyer , C. Kim , S. Y. Lee , W. G. Kim , M. Zemla , M. Auer , A. Hexemer , S. W. Lee , Nat. Commun. 2014, 5, 3043. 24448217 10.1038/ncomms4043

